# Using Heuristic Value Prediction and Dynamic Task Granularity Resizing to Improve Software Speculation

**DOI:** 10.1155/2014/478013

**Published:** 2014-05-20

**Authors:** Fan Xu, Li Shen, Zhiying Wang, Bo Su, Hui Guo, Wei Chen

**Affiliations:** National University of Defense Technology, Changsha, Hunan 410073, China

## Abstract

Exploiting potential thread-level parallelism (TLP) is becoming the key factor to improving performance of programs on multicore or many-core systems. Among various kinds of parallel execution models, the software-based speculative parallel model has become a research focus due to its low cost, high efficiency, flexibility, and scalability. The performance of the guest program under the software-based speculative parallel execution model is closely related to the speculation accuracy, the control overhead, and the rollback overhead of the model. In this paper, we first analyzed the conventional speculative parallel model and presented an analytic model of its expectation of the overall overhead, then optimized the conventional model based on the analytic model, and finally proposed a novel speculative parallel model named HEUSPEC. The HEUSPEC model includes three key techniques, namely, the heuristic value prediction, the value based correctness checking, and the dynamic task granularity resizing. We have implemented the runtime system of the model in ANSI C language. The experiment results show that when the speedup of the HEUSPEC model can reach 2.20 on the average (15% higher than conventional model) when depth is equal to 3 and 4.51 on the average (12% higher than conventional model) when speculative depth is equal to 7. Besides, it shows good scalability and lower memory cost.

## 1. Introduction

Exploiting potential thread-level parallelism (TLP) is becoming the key factor to improving performance of programs on multicore systems [1]. A series of productions provide effective solutions to parallel programming, such as OpenMP [2], MPI [3], and TBB [4]. However, as the processor cores increase and software application becomes more and more diverse, the traditional parallel programming frameworks are facing new challenges. First, the complexity of dependencies makes the program code hard to be parallelized effectively by traditional parallel programming tools. Programs with lots of conflict variables (CVARs, the variables involved in cross-iteration dependencies) usually cannot be parallelized smoothly. To solve this problem, some parallel programming tools offer explicit synchronization and communication interfaces for programmers, but this will increase the difficulty of parallel programming. Second, traditional parallel programming tools cannot support multiple parallelism modes. For example, OpenMP can support DOALL mode well but lacks support for DOACROSS or PIPELINE mode [5]. Third, as the processor core number increases, the scalability of traditional parallel programming methods faces additional challenge, too.

Speculative parallel execution model offers a solution to the problems above. It offers underlying hardware or software for correctness checking so that the programming interface is simpler. Programmers using Transactional Memory (TM) [6–9] or Thread Level Speculation (TLS) [10–14] models do not have to know the details about the dependencies between threads. They can neglect the dependencies while they are parallelizing the program and focus on the algorithm optimization or task partition. The underlying hardware or runtime system will help them to insure the program against errors. Speculative parallel model can drastically exploit parallelism in the program and reach a high performance, without increasing burden of programmers. The Stanford Hydra [15] with its TLS mechanism and various kinds of transactional memories are typical works of speculative parallel execution models.

Although the speculative parallel model is of high efficiency and practicability, there are defects of its mechanism. For conventional hardware supported models, the changes in microarchitecture are costly and less scalable. To avoid these problems, many researches on the speculative parallel models are based only on software in recent years. A series of works such as BOP [16, 17], CorD [18, 19], and SpiceC [5] are proposed and the evaluation results of them are quite good. However, for the software-only speculative parallel models, there are still two kinds of obstacles. First, the missing of hardware support usually leads to both higher control overhead and rollback overhead. Second, the static task partitioning leads to the imbalance of the loads of each speculative thread. To overcome these obstacles, special strategies are needed to reduce the overall overhead and balance the load.

Aiming at the defects in the software-only speculative parallel models, in this paper, we try to use a novel value prediction scheme and a dynamic task partitioning scheme to improve the conventional models. The paper proposes our new software-based speculative parallel model, called HEUSPEC. Two main contributions are included.

- The model uses heuristic value prediction (HVP) mechanism to reduce the high misspeculative rate in the conventional speculative parallel models. The mechanism can generate predicted values of CVARs via multiple approaches, including history value prediction scheme. It uses a scorekeeper to evaluate and select the prediction results. The mechanism can improve the accuracy of speculative read in the model and reduce the rollback overhead.
- The model involves dynamic task granularity resizing (DTGR) mechanism. The mechanism can optimize the overhead of the model at runtime by resizing the granularity of each parallel task. It can augment the task size when the misspeculation rate is at low level and deflate the task size when the rate is high. Thus it can reduce the overall time cost remarkably.

The rest of the paper is organized as follows. In Section 2, the overview of the HEUSPEC model is introduced. In Section 3, the key techniques are proposed in detail. The implementation of the model is proposed in Section 4. The evaluation and experiment results are given and analyzed in Section 5. In Section 6 we introduce some related works. Finally in Section 7, the conclusions are given.

## 2. Overview of the HEUSPEC Model

**Figure 1:**
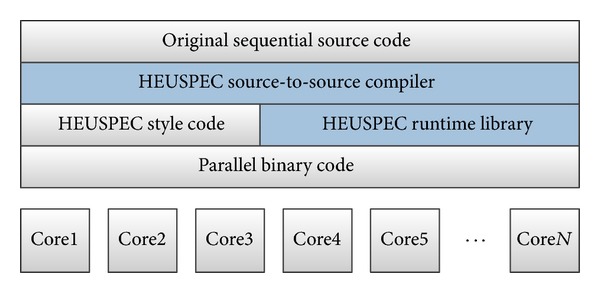
The hierarchy structure of HEUSPEC model (the shadowed parts are the HEUSPEC source-to-source compiler and the runtime library).

The HEUSPEC parallel model is a coarse-grained parallel programming framework. It consists of two parts: the runtime library and the source-to-source compiler. HEUSPEC uses two stage compiling methods.  Figure 1 shows the hierarchy structure of HEUSPEC. The programmers can parallelize the sequential program easily with HEUSPEC. First, the original source code of the sequential program is labeled by the programmer; second, programmer uses the HEUSPEC source-to-source compiler to transform the labeled code into parallel code, with multiple parallel functions implemented in the HEUSPEC runtime library. Finally, the parallel code is compiled by a normal compiler and transformed to the parallel binary code.

**Figure 2:**
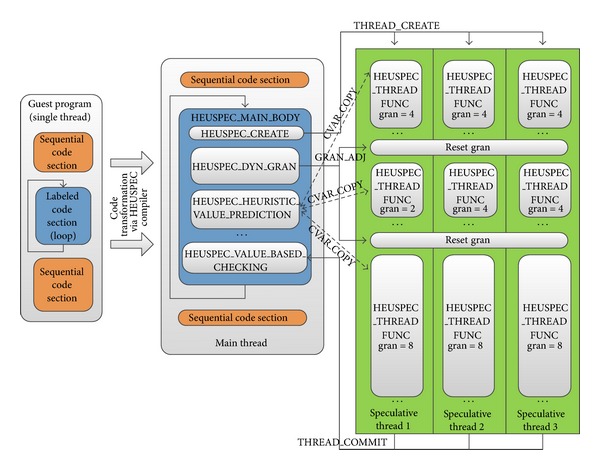
The abstract code structure of HEUSPEC model (between the main thread and speculative thread, there are several kinds of message shown in the figure, such as CVAR_COPY for copying and CVARs and GRAN_ADJ for granularity resizing. The details of the HEUSPEC message are given in Section 4).

The HEUSPEC abstract code structure is shown in Figure 2. The HEUSPEC model has one main thread and multiple speculative threads. The main thread executes the HEUSPEC_MAIN_BODY, which includes several control modules handling management work, such as speculative threads creating, CVARs management, and correctness checking. All the speculative threads are created by the main thread, which run the HEUSPEC_THREAD_FUNC code. There is no communication between the speculative threads. However, a speculative thread can communicate with the main thread via HEUSPEC messages during the speculative reading and correctness checking. As the main thread occupies a processor core during the execution, the upper bound of the speedup of HEUSPEC on an *N*-core platform is *N* − 1.

For the CVARs involved in dependencies between iterations, HEUSPEC uses the software state isolation mechanism. This mechanism is applied by the CorD [19, 20] parallel model proposed by Tian et al, which is proposed by Tian et al. in 2008, Riverside. Under this mechanism, each CVAR has a committed version and multiple speculative versions. The committed version is stored in the committed memory space, which can only be accessed by the main thread. The speculative versions are generated by the speculative threads and stored in their own private space when they start. Meanwhile, for each CVAR, the mapping relations between the committed version and speculative versions are also created by the speculative thread and recorded in the mapping table, which can be searched by main thread during the correctness checking. The speculative threads can access the speculative versions of CVARs directly in their own private space. The access trace of each speculative thread is recorded in the Read Mapping Table or Write Mapping Table. The initial value of a speculative version of a CVAR is generated by the speculative read mechanism in HEUSPEC (see Section 3.1).

**Figure 3:**
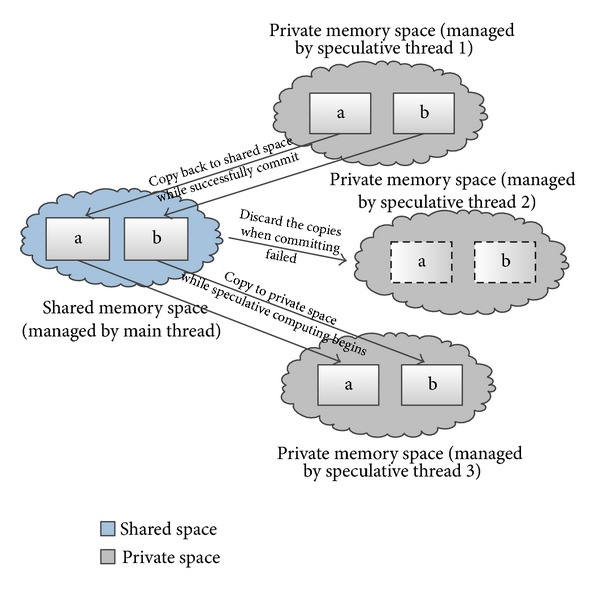
The state isolation mechanism (in the figure, we assume that the speculative thread 1 has successfully committed, the speculative thread 2 has failed during committing, and speculative thread 3 has just initialized. Therefore, the figure shows the functions of state isolation mechanism in 3 different cases, namely, copying back CVARs to shared space, discarding the CVAR copies, and copying CVAR to private space).

Figure 3 shows the state isolation mechanism in HEUSPEC. We assume that two CVARs in the code section, a and b, are copied to the private space when the speculative threads start. During the parallel execution, the speculative threads can read or modify the speculative version of a and b stored in their own private space, while the committed version is protected in the shared space. When the computing is finished, the speculative threads send messages asking the main thread for correctness checking. The main thread checks the correctness of each speculative read operation by searching in Read Mapping Table and Address Mapping Table. If there is no misspeculation, the main thread copies each modified speculative version of CVAR in the private space back to the shared space and overwrites its committed version. Or else, the speculative thread rerolls and the speculative versions of CVARs are invalidated.

HEUSPEC adopts dynamic task assignment. For example, if the labeled code section is a loop with *N* iterations, the main thread at runtime packs several successive iterations into a task and assigns the task to an idle speculative thread. When all the tasks are finished, the main thread confirms that the speculative parallel section is finished and terminates all the speculative threads. Though dynamic task assignment introduces some additional control overheads, it enables the main thread to adjust the granularity of the task at runtime and eliminates unnecessary interim thread creating and killings processes. Therefore, it definitely benefits the overall performance. We proposed dynamic task granularity resizing (DTGR) mechanism based on the dynamic task assignment (see Section 3.3).

To insure the correctness of speculative parallel execution, the speculative parallel model must include a commit mechanism (or conflict detection mechanism), so that the correct task can be committed, and the failed task can be rerolled. Most of the conventional speculative parallel models apply version based correctness checking mechanism. Under this mechanism, each copy of CVAR has its own version number: speculative version numbers for the speculative versions and committed version numbers for the committed versions. The speculative version number can be modified during a speculative writing to the CVAR. While the committed version number can only be modified while a task commits successfully. During the correctness checking, for each CVAR, the speculative version number recorded in the RAT will be compared to the current committed version number, so as to determine the correctness of the task.

In HEUSPEC, to support HVP, we must change the conventional version based correctness checking mechanism to the value based correctness checking. This mechanism was applied in the BOP [16, 17] proposed by Ding et al. in 2007 to reduce some avoidable rollback caused by written but not changed CVARs. The key idea of the value based correctness checking is that during the correctness checking, for each CVAR, the values instead of the version numbers of the speculative versions are compared to the value of the committed version to determine the correctness of a speculative task during the correctness checking. To support this, some changes in the structures of global tables (Address Mapping Table, Read Mapping Table, and Write Mapping Table) must be applied. For example, the version number fields in the tables are replaced by the size of CVAR fields. And additional memory space is required to store the speculative versions generated by HVP. For the details about the global tables, see Section 4.1.

## 3. Significant Techniques in HEUSPEC

The conventional software-based speculative parallel models are diversified in the implementations of conflict detecting and conflict solving mechanisms. However, from most models, the 3 factors that affect the performance can be abstracted, namely, the misspeculation rate, the average rollback overhead, and the average controlling overhead. The relationship between the global overhead and the 3 factors is as follows:

(1) Oglobal=Ntask×(OControl In⁡ Average+Rmiss×ORollback In⁡ Average).

The variables used in the equation are shown as follows:

- *O*_global_: global overhead of the model,
- *N*_task_: total number of tasks,
- *O*_Control In⁡ Average_: average control overhead of each task,
- *O*_Rollback In⁡ Average_: average reroll overhead of each task,
- *R*_miss_: misspeculation rate.

From (1), we can conclude that there are 3 ways to reduce *O*_global_, namely, to reduce *O*_Control In⁡ Average_, *O*_Rollback In⁡ Average_, or *R*_miss_. In fact, there is tradeoff between the 3 factors; take CorD as an example; it uses precomputing to reduce *R*_miss_ and checkpoint mechanism to reduce *O*_Rollback In⁡ Average_; however, both of them increase the *O*_Control In⁡ Average_ remarkably. In the HEUSPEC model, we proposed 2 key technologies to overcome the defects in the conventional models. The heuristic value prediction (HVP) mechanism can reduce the high *R*_miss_ in the guest program with low cost, and the dynamic task granularity resizing mechanism manages to balance the *O*_Control In⁡ Average_ and the *O*_Rollback In⁡ Average_ in order to optimize the *O*_global_.

### 3.1. Heuristic Value Prediction

**Figure 4:**
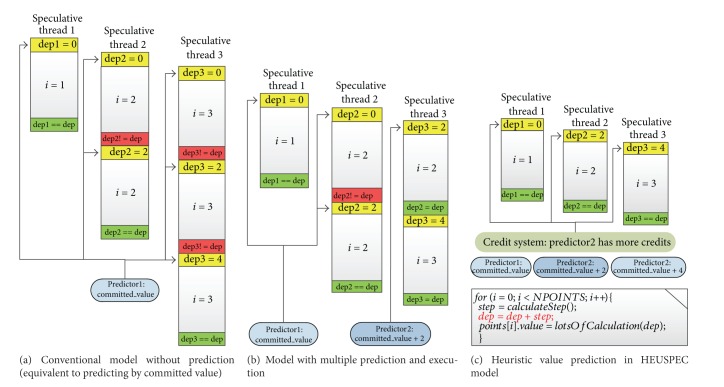
Prediction scheme in 3 different kind of models. (The code section is shown in (c). The bold line is the sentence that causes the dependency.)

The misspeculation rate (*R*_miss_) is tightly correlated with the global overhead. However, conventional speculative parallel models without value prediction have high *R*_miss_ while executing a loop with dependencies. Take the code section in Figure 4(c) as an example; the loop in the figure has lots of potential parallelism; however, a CVAR* dep* is within the loop. Using conventional model, we assume that* dep_privateN* is the speculative version of CVAR* dep* in the speculative thread *N*. If there is no explicit synchronization, the conventional model always copies the value of committed* dep* in the shared space when generating* dep_privateN*. This will cause many conflicts, make the task reroll frequently, and impact the performance of paralellized code section seriously.

To solve the problem, some previous works adopted value prediction schemes [18, 20]. However, most of them use random algorithm correlated with multiple execution (more than one processor to execute the same loop iteration) scheme in the value prediction, which is processor-consumptive and lowers down the upper limit of the overall speedup. In this paper, we try to find an effective and less processor-consumptive way to lower the *R*_miss_, hoping that the speculative read mechanism can be more “rational,” that is, to predict the value validly with some information such as loop index or history values rather than predict blindly. Therefore we proposed heuristic value prediction (HVP). We add a group of value predictors in the conventional model. Just as Figure 4(c) shows, for a single CVAR, each predictor predicts its value by a specific rule. A credit system is created to evaluate the “validity” of all the predictors. The speculative thread always adopts the value from the predictor with more credits.

The effectiveness of the HVP depends on two aspects. The first aspect is the predictability of the CVARs. If the value changing trace of a CVAR follows a specific rule potentially during the sequential execution, the variable is considered to be predictable. The second aspect is that whether the rule matches a specific predictor. If they are matched, the predictor will probably pass the correctness checking. Therefore, for those CVARs whose values change randomly, the HVP hardly improves the *R*_miss_. However, for those predictable CVARs, the mechanism can reduce the *R*_miss_ remarkably. For example, assume that the function* calculateStep()* in the code section in Figure 4(c) always returns 2; the value of the CVAR* dep* presents in a linear form. Therefore, a simple linear predictor can match it, and the pitfall of rollback introduced by* dep* will be reduced considerably, just as Figure 4(c) shows.

To apply the HVP, two hypotheses should be proved. First, there are quite a number of “predictable” CVARs in the practical applications. Second, the values of these CVARs can be predicted by some simple methods with low overheads, so that the prediction will not increase the overhead of the model too much. To prove them, we carried through an investigation to a series of applications. We found the CVARs and categorized them by their value changing rules.

**Figure 5:**
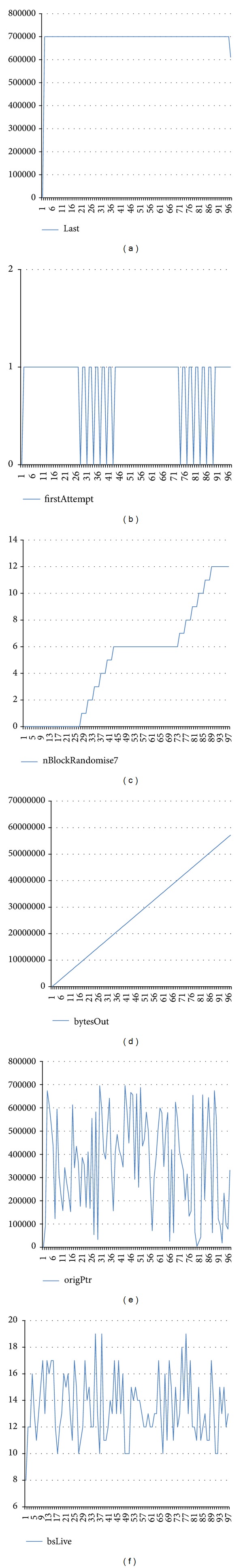
6 different patterns of CVAR's value changing.

Figure 5 shows typical examples of 6 categories. The example variables are selected from a loop in the benchmark* 256.bzip*. The loop has 97 iterations. Each subgraph shows the value changing trace of a single CVAR during the loop execution. Generally speaking, variables with random value changing traces are hard to predict, and the variables in the CONSTANT, BOOLEAN, LADDER, and LINEAR category are easier. In the selected benchmarks in our investigation, the CVARs of the last two categories (RANDOM and RESTRICTED RANDOM) account for about 19%; the rest are of the former four categories (CONSTANT, BOOLEAN, LINEAR, and LADDER). Obviously, for the CONSTANT and the LADDER category, the conventional mechanism which uses the committed value of the CVAR is the best. The BOOLEANs can be predicted with the mechanism similar to the Branch Predicting Buffer in the microprocessors. For the LINEARs, if the trace of their value can be learned, they can be predicted precisely by linear extrapolation.

Through this investigation, we can get the basic ideas of the HVP. First, among all the CVARs, there are several “predictable” CVARs, whose values are changing regularly in the loop. Second, a predictable CVAR's value can be predict through a low cost way with their history value, such as linear prediction or bool prediction. Third, the changing rule of the value of a predictable CVAR is probably steady in a period. Based on these three ideas, we developed the HVP. First, we build a group of predictor, in each of which implemented a low cost prediction way. Once a speculation read (a speculative thread reading a CVAR's value, may cause a misprediction) happens, each predictor generates a speculative value of the CVAR. One of these values will be selected as the result of the speculative read. As the changing rule of the value is probably steady, sometimes the value of a CVAR may be “catched” by a certain predictor; therefore, we can use a mechanism like scoreboard to evaluate which predictor is most probably matching the CVAR. This mechanism is called the “credit system” which is described in Section 3.2.

#### 3.1.1. HEUSPEC Predictors

**Figure 6:**
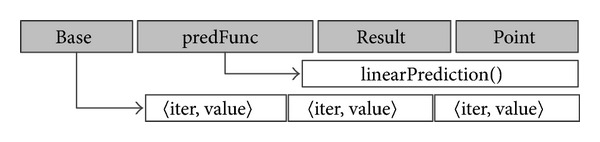
The structure of a HEUSPEC predictor (a single predictor includes four fields, namely, the* Base* field which points to an array storing the information used in prediction, the* predFunction* field which is the entry of the prediction function, the* Result* field which stores the prediction result, and the* Points* field which records the times of correct speculation the predictor has made. The elements in the* Base* array and the* Result* field are 〈iter, value〉 pairs. The figure shows a linear predictor. The size of* Base* array is 3, and the* predFunc* points to the function* linearPrediction()*).

Based on the analysis above, we designed the HVP predictors.  Figure 6 shows the 4-field structure of a HVP predictor. During the prediction, the prediction function pointed by* predFunction* pointer generates the* Result.value* based on the information in the* Base* array and* Result.iter*. Most of the time, not all elements in the* Base* array are used. For example, for the conventional predictor and the reversal predictor, only the last committed 〈iter, value〉 pairs are used. To simplify the HEUSPEC prediction mechanism, the max element number of the* Base* array is 3.

Table 1 shows all the predictors implemented in the HEUSPEC model. Among these 5 predictors, the conventional predictor inherits the speculation mechanism in the conventional models, which always uses the committed value in the shared space. The reverse predictor is for the Bool type CVARs. In the scheme of the reverse predictor, we assume that the value of the CVAR always reverses between two adjacent iterations. Therefore, the predictor can calculate the speculative value in the current iteration. For example, if the* Base[0]* is 〈1,1〉 which means that the number of the last committed iteration is 1 and the predicted value is 1 and the current iteration number is 2, the* Result* should be 〈2,0〉. The restricted random, linear, and guadratic predictors are for integers. The linear and quadratic predictors take the elements in the* Base* array as a series of points in 2-D space and use them to generate the* Result* via extrapolation method. The restricted random predictor uses the* Base* array to record the upper and lower limit.

#### 3.1.2. Credit System

The commonness of the 5 predictors is that they use, more or less, the history values of the CVARs to guide predictions. However, a single predictor has little probability to make a correct prediction. To augment the probability, the credit system is applied. For a speculative read, each predictor produces a candidate value.  Figure 6 shows the structure of the predictors. The* Points* field in each predictor records the correct speculation it has made. Through this, the credit system can quantify the “rational” level of the predictors, and select an appropriate speculative value among them.

**Figure 7:**
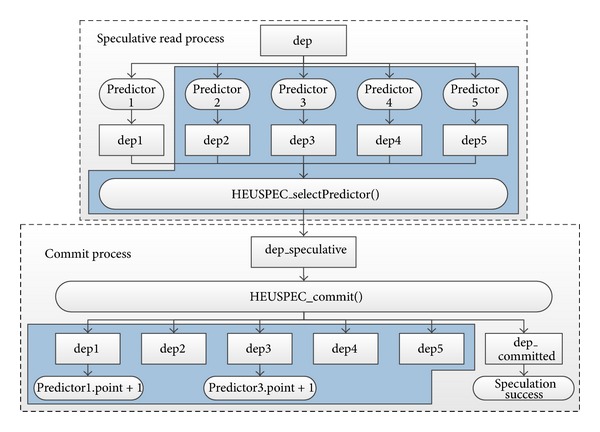
The speculative read and commit processes of heuristic value prediction. (We use the code section in Figure 4. The shadowed part of the figure shows the new workflow introduced by the heuristic value prediction. The unshadowed part is the original speculation-commit workflow of the conventional speculation model.)

Figure 7 shows the workflow of the credit system. During the speculative read process, each predictor generates a speculative value candidate (*depN* in the figure) for* dep*. Only the value generated by the predictor with the highest point is selected via the select function HEUSPEC_selectPredictor(). If multiple predictors have the same highest point, the select function selects one from them randomly. Once the speculative value is selected, it is returned to the speculative read function and used in the calculation of the speculative thread. Finally During the commit process, the commit function* HEUSPEC_commit()* compares all the generated speculative value, no matter used or not, with the committed value in the shared space. If a predictor did a correct prediction, it gains an additional 1 point. Therefore, the HVP can generate speculative values of CVARs more rationally, making use of their history values.

Although the HVP cannot insure that the predictions are always correct, it can improve the speculation accuracy by a certain extent. Especially for those CVARs whose values changing pattern matches a given predictor, most of the rollback can be eliminated. The evaluation and result of HVP are discussed in Section 5.1.

### 3.2. Dynamic Task Granularity Resizing

**Figure 8:**
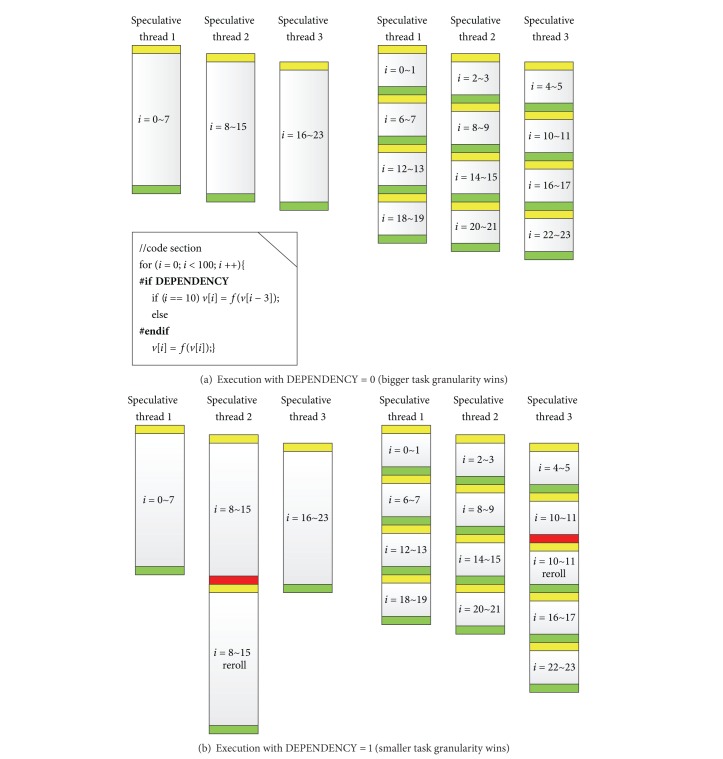
The execution flow of a code section under the conventional speculative model with static task granularity (the two parts of the figure share the same code section, in which the “#ifdef” part in the code section brings a dependency. The shadowed part in the task shows the control overhead brought by task creating and committing. The (a) part of the figure shows the execution flow with DEPENDENCY = 0, while the (b) part shows the execution flow with DEPENDENCY = 1. The bigger task granularity is 8, while the smaller task granularity is 2).

The Dynamic task granularity resizing (DTGR) is intended to reduce the global overhead of the HEUSPEC model and balance the load. Models with static task granularity always suffer from much additional overhead and imbalanced load. As Figure 8 shows, two different code sections (DEPENDENCY = 1 or not) are executed under a conventional model with static task granularity. Obviously, for a high misspeculation rate (*R*_miss_), granularity should be lowered down to avoid additional rollback overhead. However, for a low *R*_miss_, finer tasks will break the continually of the calculation and cause a lot of control overhead, so in this case the bigger granularity is better. In fact, the appropriate granularity task is related not only to the rollback rate, but also to the computation in the parallel section and control overhead caused by task creating and committing. Therefore, to find the appropriate granularity, we use the dynamic optimization technique.

We believe that the global speculation overhead can be optimized to adapt the runtime behavior of a program via dynamic optimization. In Section 3, we have created the analytical model of global overhead in the HEUSPEC, which is described by (1). We have analyzed that the global overhead depends on the 3 factors, namely, the average control overhead (*O*_Control In⁡ Average_), the misspeculation rate (*R*_miss_), and the average rollback overhead (*O*_Rollback In⁡ Average_). In this section we give a further discussion about the *O*_global_. The variables used are listed:

- *N*_task_: total task number,
- *N*_iter_: total iteration number of the loop,
- *R*_miss_: miss rate,
- *N*_miss_: total miss time of the loop,
- *N*_thread_: total number of speculative threads,
- gran: task granularity,
- *O*_Control In⁡ Average_: average control overhead of each task,
- *O*_Rollback In⁡ Average_: average rollback overhead of each task,
- *O*_Rollback  *Per*⁡ Iter_: rollback overhead of a single iteration.

In a period, if task granularity is constant, we have *N*_task_ = *N*_iter_/gran, *R*_miss_ = *N*_miss_/*N*_task_, and *O*_Rollback In⁡ Average_ = gran × *O*_Rollback *Per*⁡ Iter_; therefore, we can transform (1) as follows:

(2) Oglobal=(Nitergran)×(OControl In⁡ Average+(Nmiss(Niter/gran))  × gran×ORollback Per⁡ Iter).

Equation (2) can be simplified as follows:

(3) Oglobal=(Nitergran×OControl In⁡ Average)+(Nmiss×gran×ORollback Per⁡ Iter).

Since gran ≥ 1, to minimize the GO, we derive both sides of (3) by gran. Therefore we have

(4) Oglobal′(gran)=(−Niter×OControl In⁡ Average×1gran2)+(Nmiss×ORollback Per⁡ Iter).

Let *O*_global_′(gran) = 0; if *N*_miss_ ≠ 0, we have

(5) $$\begin{array}{c} \text{gran}=\sqrt{\frac{\left(N_{\text{iter}}\times O_{\text{Control}\;\mathrm{In}\;\text{Average}}\right)}{\left(N_{\text{miss}}\times O_{\text{Rollback}\;\mathrm{Per}\;\text{Iter}}\right)}}\;\left(N_{\text{miss}}\neq 0\right), \end{array}$$

From above analysis, we can make conclusion. If we take a certain number of iterations as an adjusting period (AP), we optimize gran according to (5). During an adjusting period, the *N*_iter_ is a constant, while the *O*_Control In⁡ Average_, the *O*_Rollback *Per*⁡ Iter_, and the *N*_miss_ can be calculated by a group of profiling counters at runtime. Therefore, we can calculate the optimized gran dynamically. However, since the *N*_miss_ can be zero and the calculation may fail, in that case, we prescribe that when *N*_miss_ = 0, we let gran = *N*_iter_/*N*_thread_. *N*_thread_ represents the number of speculative thread.

**Figure 9:**
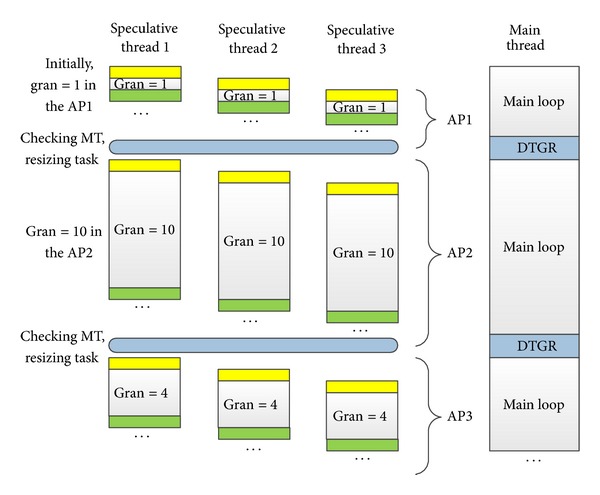
Dynamic task granularity resizing (in the speculative thread columns, the shadowed parts represent the control overheads).

In the HEUSPEC, the DTGR is implemented as a module embedded in the HEUSPEC_MAIN_BODY (shown in Figure 2), which is executed by the main thread. Initially, we set gran to a certain value (in the experiment in Section 5, it is 1) then recalculate and update the gran at the beginning of every adjusting period. After that, the succeeded tasks are assigned with the granularity equal to the newly updated gran. The DTGR assures that tasks are assigned betimes to idle speculative threads.  Figure 9 shows the details of DTGR. We let a constant number of iterations be an adjusting period (AP). The main thread executes task granularity resizing function to adjust the task granularity with dynamically calculated *N*_iter_, *O*_Control In⁡ Average_, *N*_miss_, and *O*_Rollback *Per*⁡ Iter_. After resizing, the main thread assigns task with new granularity.

Through the DTGR, programmers are able to optimize the global overhead and balance the load without considering the task partition scheme. According to our experiment, however, some profiling information such as *O*_Control In⁡ Average_ and *O*_Rollback *Per*⁡ Iter_ can just reflect the average behavior of the loop iterations in a constant time period. Therefore, this method can assuredly help the programs without much computation difference across iterations (such as the loops in* MatMul* or* LU*). For those loops with large across-iteration computation difference, we cannot ensure that the granularity will have converge to the best value. But this method includes the dynamic assignment, with which the performance loss can be made up, and we still can get an acceptable speedup. The details of the experiment results and analysis are given in Section 5.

### 3.3. Other Optimizations

Besides the two key techniques, we have adopted some other optimizations. The out-of-order confirming mode is used for those benchmarks without dependencies. Because there is no dependency between the adjacent speculative threads, thus the implicit synchronization in the in-order confirming mode can be eliminated. The on-the-fly copying is adopted for those read-only CVARs. If a CVAR is read-only in the parallel section, it is not necessary to generate its speculative version. Instead, we can use the committed version directly. The out-of-order confirming mode and on-the-fly copying are adopted in the HEUSPEC model, in order to further reduce its time and memory cost.

## 4. Implementation

As Figure 1 shows, the HEUSPEC includes a source-to-source compiler and a runtime library. We implemented the compiler via LLVM framework and the runtime library via POSIX thread library in ANSI C. The HEUSPEC model is implemented based on the traditional software-based speculative model. The two key techniques proposed in Section 3 are implemented in the runtime library. In this section we show details of the global tables and the implementation of the HEUSPEC.

### 4.1. Global Tables

**Figure 10:**
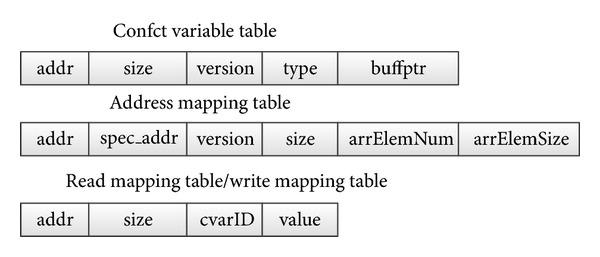
The structures of the global tables (there are only one Conflict Variable Table. The number of Address Mapping Tables and Read Mapping Tables/Write Mapping Tables is equal to the *N*_thread_).

We have introduced in Section 2 that the state isolation mechanism is the basic mechanism of the HEUSPEC model. To manage the different versions of CVARs, it is necessary to create the mapping relations between the speculative versions and committed version for each CVAR. Meanwhile, the access information should be recorded, too. Therefore, the sufficiency of the information for the correctness checking and task committing can be insured. In the HEUSPEC model, the information related to speculative execution is stored in several global tables.  Figure 10 shows the structures of the global tables.

 The* Conflict Variable Table*, which can only be accessed by the main thread, is used to store the basic information of the CVARs, such as address (for the committed version), size, or type. For each CVAR, there is a record in the CVAR Table.
 The* Address Mapping Table* is used to maintain the mapping relation between the addresses of committed version (*addr*) and the speculative version (*spec_addr*). Each speculative thread has its own Address Mapping Table.
 The* Read Mapping Table* and* Write Mapping Table* are used to keep the record of accesses to the CVARs for each speculative thread. The* value* field in the two tables is used to store the speculative value of the CVARs while being read or written. This value will be used in the committing process.

### 4.2. HEUSPEC Style Code

The HEUSPEC source-to-source compiler can translate the labeled C code into HEUSPEC style C code. During this process, the original C code is transformed, mixed with HEUSPEC runtime library function, and finally transformed to the code which can be parallel executed. In Figure 2 we have shown the abstract code structure of the HEUSPEC code. Algorithm 1 shows an example of the code transformation of a real benchmark (*Pi* in the OmpSrc 2.0).

## 5. Experiments and Evaluation

To test the performance of the HEUSPEC model and prove its advantage comparing with the conventional model, we designed and carried on a series of experiments. We choose the hardware platform with two Xeon5450 processors, which have 4 processor cores. The capacity of the memory is 24 GB. The software environment includes a Linux OS (kernel version 2.6.32) and a C compiler. We chose the benchmarks of different CVAR numbers.  Table 2 lists the benchmarks we used in the experiments.

We have designed four experiments to test the performance of the HEUSPEC. First, to show how speculation accuracy improved by HVP; we did the experiment and gathered the miss rate with two typical benchmark on multiple levels of task granularity. Second, to reflect the performance gain by the HEUSPEC, we have contrasted the performance speedup of a program executed under the HEUSPEC against that under the conventional model. Third, to reflect the scalability of the HEUSPEC, we have tested the speedup of each benchmark under the HEUSPEC as the speculative depth (the number of concurrent speculative threads) increases. Forth, we have tested the control overhead introduced by the HEUSPEC.

### 5.1. Speculation Accuracy Improvement

We choose the benchmark* badloop* and * fluidanimate* and run them under the HEUSPEC. To show the miss rate improvement with different task granularity levels, we shut down the DTGR. For* badloop*, we choose 6 levels of task granularity, and for* fluidanimate*, we choose 3 levels of task granularity.

**Figure 11:**
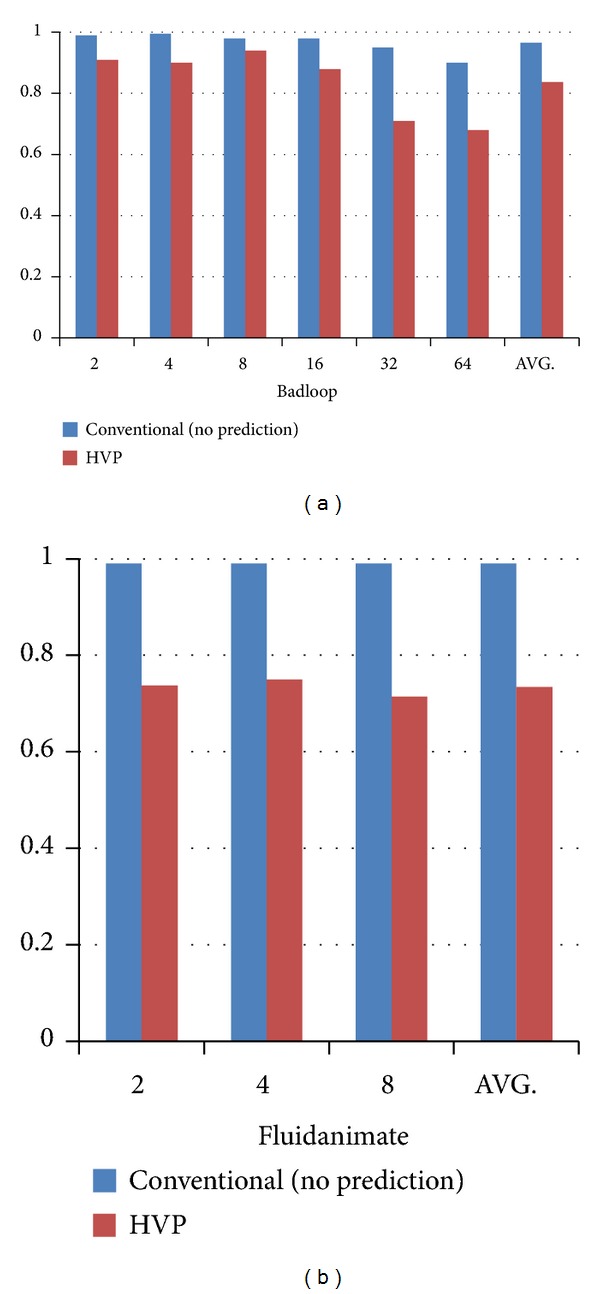
The miss rate under HEUSPEC with HVP.

Figure 11 shows the experiment result: the miss rate of badloop reduced by 12.9% on the average and the miss rate of fluidanimate reduced by 25.6%. The experiment shows that the HVP actually reduced the miss rate of speculation.

### 5.2. Speedup

**Figure 12:**
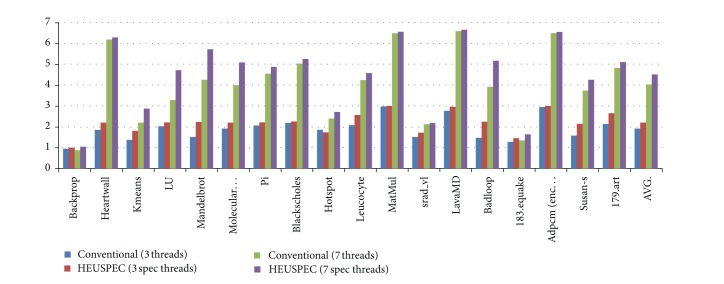
The speedup of HEUSPEC against conventional model. (We show the speedup under the conventional model with 3 speculative threads and the speedup under the HEUSPEC with 3 and 7 speculative threads, resp.)

Figure 12 shows the speedup of each benchmark under the HEUSPEC against that under the conventional model. In this experiment, we run the benchmarks under the conventional model (without HVP and DTGR) and the HEUSPEC, respectively. We used out of order confirm mode for* MatMul*,* lavaMD*,* adpcm*, and* blackscholes*, thus greatly improving the performance of those benchmarks without dependencies. According to our experiment, when the speculative depth is 3, the average speedup of the HEUSPEC is 2.20, about 15% higher than that of the conventional model(1.91). The speedup of the HEUSPEC can reach a high level when the speculative depth is 7, about 4.51 on the average, about 12% higher than that of the conventional model(4.02).

On one hand, the HVP aims at the cross-iteration dependencies of the loop. Therefore, it is more efficient on those benchmarks which have more predictable CVARs. On the other hand, a loop with bigger iteration number and intensive computation can introduce a larger space of optimization for DTGR. Therefore, the DTGR prefers the benchmarks with this feature. From Figure 12, we can see that some benchmarks (*badloop*,* LU*,* Molecular Dynamic*,* Mandelbrot*, and* kmeans*) show remarkable improvement compared with the conventional model (especially with 7 speculative threads). That is mainly because they fit the two conditions we mentioned above. Some benchmarks (*heartwall*,* blackscholes*,* hotspot*,* leucocyte*,* MatMul*,* lavaMD*, and* adpcm*) show a high speedup compared with the serial execution, but little improvement compared with the conventional model. That means that HVP and DTGR are less efficient in these benchmarks, because they have little predictable CVAR, and less optimized space for DTGR. Some other benchmarks (backprop, srad_v1, and 183.equake) have low speedup compared with other benchmarks. That is mainly because the computation in the parallelized loop in these benchmarks is not enough, and the global overhead of HEUSPEC is too much for them.

Due to the unavoidable rollback overhead or the high control overhead, several benchmarks show low speedups under the speculative parallel model, such as* backprop*,* 183.equake*, and* srad_v1*. However, most of the benchmarks show remarkable performance gain on this experiment.

### 5.3. Scalability

**Figure 13:**
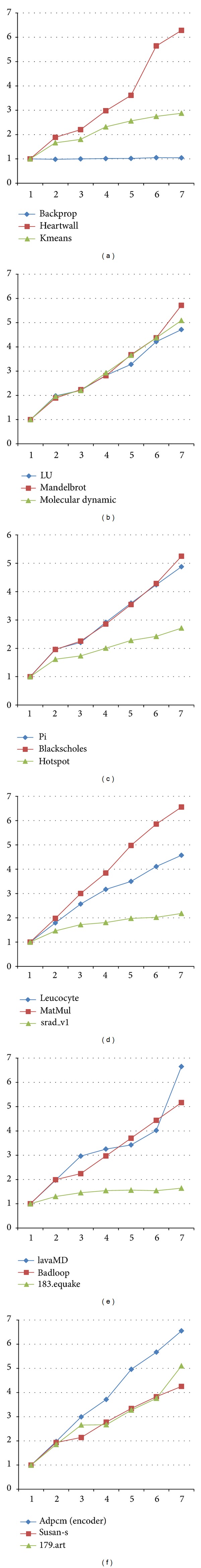
The speedup of the benchmarks under HEUSPEC with the speculation depth changes from 3 to 7.

Figure 13 shows that the speedup improved along with the speculative depth increases under the HEUSPEC, which can reflect the scalability of the HEUSPEC model to a certain extent. In our experiment, the performances of all the benchmarks improve as the speculation depth increases. Among them,* adpcm* and* MatMul* show better scalability, while some other benchmarks show worse, such as* backprop* or* 183.equake*.

The speedup of a benchmark depends on two factors. On the one hand, the rate of the parallel section is relative to the speedup of the benchmark. For example, the parallel section of the* MatMul* benchmark accounts for more than 97% code, while the parallel section of the* backprop* benchmark accounts for less than 30%. Thus the former shows better speedup and scalability than others while the latter performances are worse. On the other hand, the code structure of the parallel section can also influence the speedup. For example, the parallel section of the benchmark* 183.equake* is in the function* smvp_opt()*; this function is repeatedly called in another loop in the* main()*. This makes the program call the* HEUSPEC_main_body()* repeatedly, bringing much control overhead. Therefore, it shows a bad speedup and scalability.

### 5.4. Time and Space Overhead

**Figure 14:**
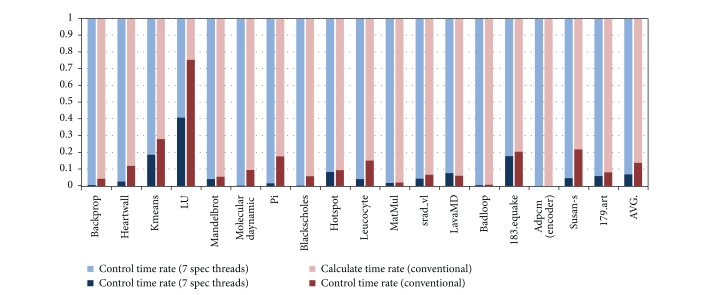
The control overhead introduced by the HEUSPEC model. (The benchmarks with higher control overhead is mainly due to the repeated calling of HEUSPEC_main_body(), such as LU, kmeans and 183.equake.)

Figure 14 shows the control overhead introduced by the HEUSPEC. The control overhead includes the time cost on the CVAR copy, task creating and eliminating, correctness checking, and communication between speculative threads and main thread. The experiment is carried with the speculation depth equaling to 7. We compared the result with that of the conventional model. The overall control overhead is 6% on the average, about 7% lower than that of the conventional model. Except several benchmarks such as LU, kmeans, and 181.equake with higher control overhead, for most benchmarks, the control overhead is lower than 4%.

**Figure 15:**
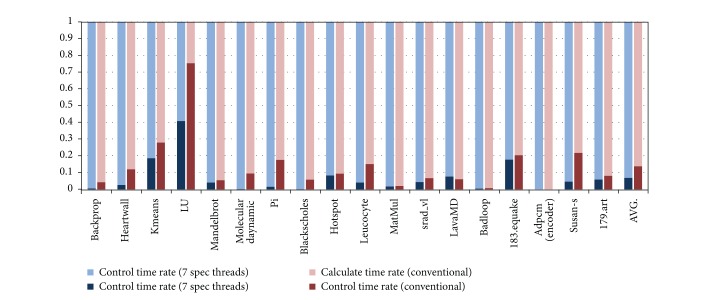
The additional memory cost introduced by HEUSPEC. (The experiment is done with 7 speculative threads. The increasing rate of the memory cost is about 21% on the average.)

Figure 15 shows the additional space overhead introduced by HEUSPEC. We carried on this experiment with 7 speculative threads and used on-the-fly copying to reduce the memory cost further. According to our experiment results, the average memory cost increased by 21% on the average under the HEUSPEC. Compared to other software-based speculative models, the additional space overhead of the HEUSPEC is much lower.

## 6. Related Works

The hardware based speculation model has not been widely used due to its limited availability. The researchers concentrated on software speculation mechanism in recent years. To reduce the overhead and to improve the accuracy are the key problems in the software speculation model research in recent years.

Ding et al. have proposed behavior oriented parallelism (BOP) mechanism [16, 17]. In the BOP, the UNIX process is used to encapsulate the speculative thread information. The shared variables are copied to the private space of each speculative thread when the UNIX process is forked. For each CVAR, the BOP allocates a single page to store it. Compared with traditional conflict detecting techniques, the BOP uses value based correctness checking, rather than version based checking, which can avoid some unnecessary rerolls of speculative threads and improve the overall performance. BOP supports DOACROSS parallel model through the dynamic dependence hints.

The copy or discard (CorD) [18, 19] execution model implemented by Tian et al. is another software speculation mechanism. In the CorD, the variables may have dependencies identified by compiler and “copied-in” to the private memory space (*P* space) of the speculation threads. Conflicts are detected and handled by main threads, which is a manager thread without doing any calculation. Ideally, the overall speedup using CorD can approach to *p* − 1 in a *p*-core platform. To reduce the misspeculation rate, CorD has brought in the “multiple random value prediction” mechanism, which uses 3 or more predictors to generate the speculative values of the CVARs. Under this mechanism, several processor cores are used to execute same iterations to increase the speculation accuracy. The “pre-computing” technique is also used to improve the accuracy of the speculation.

Liu et al., in University of California Irvine, have performed speculative execution with multiple value prediction on GPUs [20]. Similar to the CorD, for each CVAR, it uses multiple random value prediction mechanism. A single loop iteration may have several copies executed in different threads with different sets of predicted CVAR values. For each loop iteration and its copies, the earliest finished one which passed the correctness checking can submit, while others are discarded. This mechanism can improve the speculation accuracy remarkably with large hardware thread consumption (a task with *n* CVARs and *m* possible values for each CVAR may have *m* ^*n*^ copies and need the same number of hardware threads to execute them in parallel). With the help of GPU architecture, the number of the predictors is very large. The CVAR values generated by different predictors are mapped to different speculative threads which run in parallel, thus providing a high speculation accuracy and reducing the rollback overhead.

## 7. Conclusion

We presented a novel speculation parallel execution model: the HEUSPEC. Based on the conventional software speculation parallel execution model, the HEUSPEC adopts 2 key techniques, heuristic value prediction (HVP) and dynamic task granularity resizing (DTGR). The HVP is adopted to reduce the misspeculation rate. The DTGR is implemented to reduce the global overhead and balance the load of the speculative threads. With 18 different benchmarks and 7 speculative threads, our experiments show that the HEUSPEC achieves a speedup of 4.51 on the average (12% higher than conventional model), and 6.56 of the highest on a 8-core platform. The model also shows good scalability and low time and space overheads.

## Figures and Tables

**Algorithm 1:**
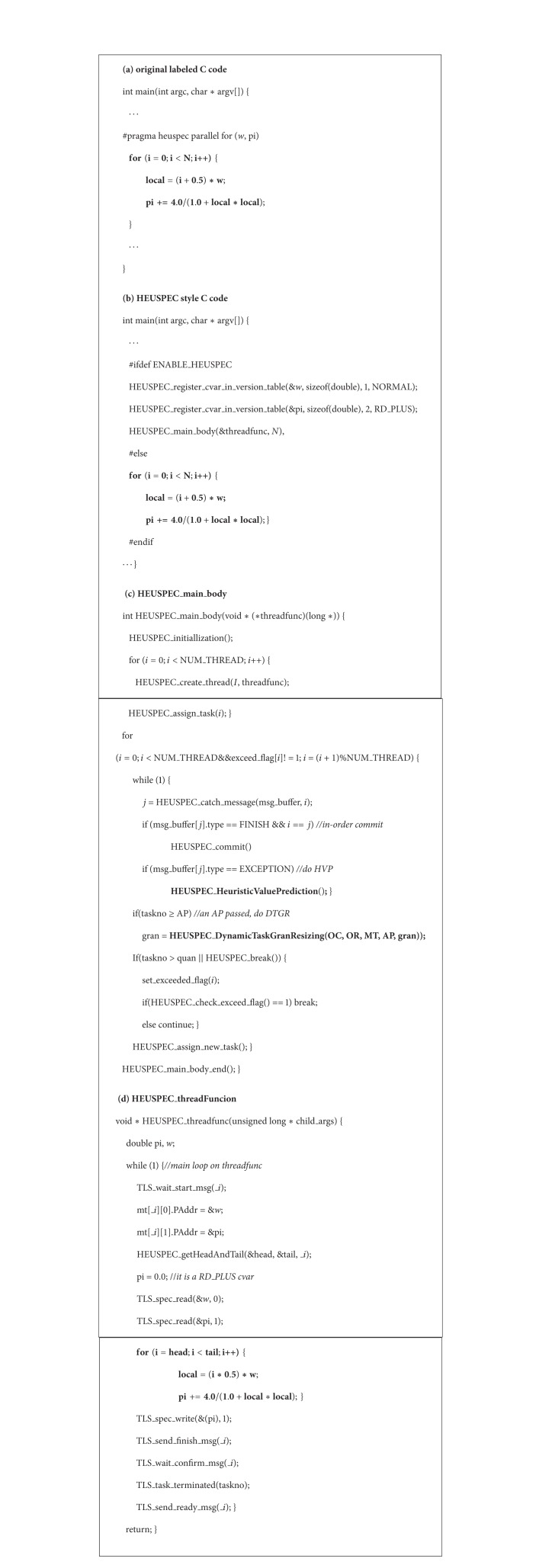
A code example optimized by HEUSPEC model. (The benchmark is Pi in the OmpSrc package. (a) the labeled sequential code. The loop is bolded. (b) the HEUSPEC style code after transformation. (c) the HEUSPEC_main_body() function. The HVP and the DTGR are bolded. (d) the HEUSPEC_threadFunc().)

**Table 1:** The HEUSPEC predictors.

Name	Base elements used	Especially applicable category	Speculative value depends on	Example
Reverse	1	BOOLEAN	The reverse of the value in the last iteration	Base[0] = 〈1,1〉 **Result =** 〈2, **R** **e** **v** **e** **r** **s** **e**(1)〉
Conventional	1	CONSTANT, LADDER	The committed value in the shared space	Base[0] = 〈1,135〉 **Result =** 〈2,135〉
Restricted random	2	RESTRICTED RANDOM	A random number in a restricted value space	Base[0] = 〈1,20〉 Base[1] = 〈2,5〉 **Result =** 〈3, **R** **a** **n** **d**(5,12)〉
Linear	2	LINEAR	The linear extrapolation of last 2 committed values	Base[0] = 〈1,20〉 Base[1] = 〈2,5〉 **Result =** 〈3, **L** **i** **n** **e** **a** **r** **E** **x** **t** **r** **a**(1,20,2, 5)〉
Quadratic	3	LINEAR, CONSTANT	The quadratic extrapolation of last 3 committed values	Base[0] = 〈1,20〉 Base[1] = 〈2,5〉 Base[2] = 〈3,8〉 **Result =** 〈4, **Q** **u** **a** **d** **E** **x** **t** **r** **a**(1,20,2, 5,3, 8)〉

**Table 2:** The benchmarks used in experiment.

Name	Package	CVAR number
backprop	Rodinia 2.1	5
heartwall	Rodinia 2.1	2
kmeans	Rodinia 2.1	8
hotspot	Rodinia 2.1	10
leucocyte	Rodinia 2.1	16
srad_v1	Rodinia 2.1	13
lavaMD	Rodinia 2.1	5
adpcm	mibench	2
susan-s	mibench	8
183.equake	Spec2K INT	5
179.art	Spec2K INT	13
LU	OmpSrc 2.0	4
mandelbrot	OmpSrc 2.0	3
MD	OmpSrc 2.0	7
Pi	OmpSrc 2.0	2
blackscholes	Parsec 2.1	0
badloop	self-coded	1
MatMul	self-coded	0
